# Survival past five years with advanced, *EGFR*-mutated or *ALK*-rearranged non-small cell lung cancer—is there a “tail plateau” in the survival curve of these patients?

**DOI:** 10.1186/s12885-022-09421-7

**Published:** 2022-03-25

**Authors:** Shoko Sonobe Shimamura, Takehito Shukuya, Tetsuhiko Asao, Daisuke Hayakawa, Kana Kurokawa, Shiting Xu, Keita Miura, Yoichiro Mitsuishi, Ken Tajima, Rina Shibayama, Naoko Shimada, Fumiyuki Takahashi, Kazuhisa Takahashi

**Affiliations:** Department of Respiratory Medicine, Graduate School of Medicine, Juntendo University, 3-1-3 Hongo, Bunkyo-Ku, Tokyo, 113-8421 Japan

**Keywords:** *EGFR* mutation, *ALK* rearrangement, NSCLC, Long survival

## Abstract

**Background:**

The prognosis of patients with NSCLC harboring oncogenic driver gene alterations, such as *EGFR* gene mutations or *ALK* fusion, has improved dramatically with the advent of corresponding molecularly targeted drugs. As patients were followed up for about five years in most clinical trials, the long-term outcomes beyond 5 years are unclear. The objectives of this study are to explore the clinical course beyond five years of chemotherapy initiation and to investigate factors that lead to long-term survival.

**Methods:**

One hundred and seventy-seven patients with advanced, *EGFR*-mutated or *ALK*-rearranged NSCLC who received their first chemotherapy between December 2008 and September 2015 were included. Kaplan Meier curves were drawn for the total cohort and according to subgroups of patients’ characteristics.

**Results:**

Median OS in the total cohort was 40.6 months, the one-year survival rate was 89%, the three-year survival rate was 54%, and the five-year survival rate was 28%. Median OS was 36.9 months in *EGFR*-mutated patients and 55.4 months in *ALK*-rearranged patients. The OS curve seemed to plateau after 72 months, and most of the patients who were still alive after more than five years are on treatment. Female sex, age under 75 years, an ECOG PS of 0 to 1, *ALK* rearrangement, postoperative recurrence, and presence of brain metastasis were significantly associated with longer OS.

**Conclusions:**

A tail plateau was found in the survival curves of patients with advanced, *EGFR*-mutated and *ALK*-rearranged NSCLC, but most were on treatment, especially with *EGFR*-mutated NSCLC.

**Supplementary Information:**

The online version contains supplementary material available at 10.1186/s12885-022-09421-7.

## Background

Globally, lung cancer cases and deaths are increasing. In 2020, GLOBOCAN estimated 2.2 million new cases (11.4% of total cancer cases) and 1.79 million deaths (18.0% of total cancer deaths), making it the most frequent cancer and cause of death due to cancer [1]. The 5-year overall survival rate for non-small cell lung cancer (NSCLC) remains poor, from 68% in patients with stageIB disease to 0–10% in patients with stage IVA/IVB disease [2]. The Surveillance, Epidemiology, and End Results (SEER) program has reported a five-year relative survival rate for unselected patients with distant-stage NSCLC of just 6.9% [3].

The epidermal growth factor receptor (*EGFR*) gene mutations were discovered in 2004 and it was reported that EGFR tyrosine kinase inhibitors (EGFR-TKIs) shrink tumors of NSCLC carrying this mutation [4]. Several phase III trials comparing EGFR-TKIs and platinum combination therapy in patients with *EGFR*-mutated NSCLC had been reported, and in all of these trials, the EGFR-TKIs groups had significantly better results in term of progression-free survival (PFS) [5–7]. In the FLAURA trial, the osimertinib group had significantly better PFS than the standard-treatment group receiving gefitinib or erlotinib [8]. Therefore, the use of osimertinib is recommended as first-line chemotherapy for *EGFR-*mutated NSCLC as well as other EGFR-TKIs with or without anti-Vascular Endothelial Growth Factor (VEGF) antibodies [9]. In the FLAURA trial, median overall survival (OS) was 38.6 months in the osimertinib group and 31.8 months in the gefitinib or erlotinib group [10], with the advent of EGFR-TKIs improving prognosis in the *EGFR*-mutated NSCLC.

Analplastic lymphoma kinase (*ALK)* is one of the receptor tyrosine kinases. The fusion gene of echinoderm microtubule-associated protein-like 4 (*EML4*) and the *ALK* gene was discovered in lung cancer cells in 2007 [11]. Its inhibitor, crizotinib, showed prominent effects in patients with *ALK-*rearranged NSCLC [12, 13]. Later, a second-generation ALK inhibitor was developed, alectinib, which inhibits ALK kinase activity more selectively. Phase III clinical trials comparing crizotinib and alectinib as the first-line chemotherapy, the so-called ALEX and J-ALEX studies, demonstrated that alectinib was superior to crizotinib in term of PFS [14, 15]. Based on these studies, alectinib is recommended as first-line chemotherapy for *ALK*-rearranged NSCLC. OS in the ALEX trial was 57.4 months in the crizotinib group and unpredictable in the alectinib group, indicating a prominent prolongation of OS.

There are not many reports showing long-term efficacy for patients with *EGFR*-mutated NSCLC, and few reports showing long-term efficacy beyond 5 years. Ten of 124 NSCLC patients survived more than five years, and these patients had Performance status (PS) 0–1 and adenocarcinoma, suggesting that good PS is a factor for long-term survival in the study published in 2010 [16]. Twenty of the 137 patients with *EGFR*-mutated NSCLC were five-year survivors in the study published in 2016 [17]. On multivariate analysis, exon 19 deletions, absence of extrathoracic or brain metastasis, and not being a current smoker were associated with prolonged OS. However, these papers included only *EGFR*-mutated NSCLC patients, and were reported several years ago with shorter follow-up periods. Immune checkpoint inhibitors (ICIs) have shown the benefit of prolonging PFS and OS with a “tail-plateau” on Kaplan Meier survival curves, and have been introduced into daily clinical practice [18]. On the other hand, there have been no reports investigating longer outcomes of more than five years or checking for the existence of a “tail-plateau” in the survival curves of patients with *EGFR*-mutated or *ALK*-rearranged NSCLC.

The primary objective of this study was to explore the survival past five years from the initiation of chemotherapy. Secondary objective was to investigate factors that lead to long-term survival in *EGFR*-mutated and *ALK*-rearranged NSCLC, in real-world clinical settings.

## Methods

### Study design and patients

Patients enrolled in this study had clinical stage III, clinical stage IV, or postoperative recurrence of *EGFR*-mutated or *ALK*-rearranged NSCLC, or radiotherapy with curative intent, and received their first chemotherapy at Juntendo University Hospital between December 10, 2008, and September 30, 2015. The presence of an *EGFR* mutations was evaluated by the Cycleave method or Scorpion-ARMS method, and *ALK* rearrangement was evaluated by a highly sensitive immunohistochemical (IHC) method or fluorescent in situ hybridization (FISH). The above criteria were met for 177 patients with *EGFR* -mutated or *ALK*-rearranged NSCLC; 155 patients had *EGFR*-mutated and 22 patients had *ALK*-rearranged NSCLC.

### Data collection

All data were retrospectively collected from electronic medical records. Patient characteristics, treatment, and outcomes were extracted. Patient-specific variables included age, sex, smoking status, Eastern Cooperative Oncology Group Performance Status Scale (ECOG PS), tumor histology, genetic mutation, staging, presence of extrathoracic metastasis, and presence of brain metastasis (Tables 1 and 3). Schedules of treatments and outcomes were also collected. The histological analysis of the tumor was based on the WHO classification. Staging was performed for all patients according to the TNM classification of the Union International for Cancer Control (UICC) ( 7^th^ edition, 2012) [19]. All patients underwent a computed tomography (CT) scan of the thorax and abdomen, a bone scintigram or positron emission tomography scan, and a brain CT or magnetic resonance imaging for TNM staging before the initiation of first-line chemotherapy, and the presence or absence of extrathoracic and brain metastases was determined. Those patients with a pleural effusion alone were not included among those with extrathoracic metastases. Databases were locked on September 30, 2020. This study protocol was approved by the Institutional Review Board of Juntendo University Graduate School of Medicine (H21-0053).

**Table 1:** Clinical characteristics of all patients

Characteristics	All		*EGFR*-Mutated		*ALK*-Rearranged	
	*N* = 177	%	*N* = 155	%	*N* = 22	%
Age						
Median (range), years	65 (34–89)		65 (34–89)		57.5 (35–77)	
Sex						
Male	82	46.3	69	44.5	13	59.1
Female	95	53.6	86	55.4	9	40.9
Smoking status						
Never-smoker	93	52.5	84	54.2	9	40.9
Current or former smoker	79	44.5	66	42.5	13	59.1
Unknown	5	2.8	5	3.2	0	0
Pack-years^a^Median (range)	26 (2–114)		27 (2–114)		17 (5–60)	
ECOG performance status						
0	96	54.2	83	53.5	13	59.1
1	54	30.5	46	29.6	8	36.4
2	11	6.2	11	7.1	0	0
> 2	10	5.5	9	5.8	1	4.5
Unknown	6	3.3	6	3.8	0	0
Tumor histology						
Adenocarcinoma	165	93.2	145	93.5	20	90.9
Adenosquamous carcinoma	4	2.2	4	2.6	0	0
Squamous	2	1.1	1	0.64	1	4.5
NSCLC, NOS	2	1.1	1	0.64	1	4.5
Pleomorphic carcinoma	1	0.56	1	0.64	0	0
Unknown	3	1.6	3	3.9	0	0
Stage						
Postoperative relapse	72	40.6	64	41.3	8	36.4
III	8	4.5	4	2.6	4	18.2
IV	97	54.8	87	56.1	10	45.4
Presence of extrathoracic metastases^b c^						
Yes	113	63.8	100	64.5	13	59.1
No	64	36.1	55	35.4	9	40.9
Presence of brain metastases^b^						
Yes	45	25.4	41	26.4	4	18.2
No	132	74.5	114	73.5	18	81,8
Treatment	All		*EGFR*-Mutated		*ALK*-Rearranged	
Number of chemotherapeutic regimens, Median	3 (1–14)		3 (1–14)		3 (1–9)	
(range)	2(0–7)		2 (0–7)		1 (0–4)	
Molecular targeted drugs	1 (0–7)		1 (0–7)		1 (0–5)	
Cytotoxic chemotherapy Immunotherapy	0 (0–2)		0 (0–2)		0 (0–1)	

^a^ pack year among Current or former smoker.

^b^ patients with radiologically proven extrathoracic or brain metastases before the start of first chemotherapy.

^c^ patients with pleurat effusion was not included in extrathoracic metastases

### Statistical considerations

Overall survival (OS) was calculated from the date of initiation of first-line chemotherapy to the date of death, and was censored at the date of the last visit for patients whose deaths could not be confirmed. Subgroup analyses of OS were performed. Descriptions of the eight subgroups are listed in Supplemental Fig. 1 and include genetic mutation, age, sex, smoking status, ECOG PS, stage, presence of extrathoracic metastases, and presence of brain metastases. OS was estimated using the Kaplan–Meier method and log-rank tests were performed to compare survival between two or three groups (Supplemental Figs. 1 and  2). Statistical significance was defined as *P* < 0.05. Hazard ratios were calculated with univariate and multivariate analysis (Cox proportional hazard model) (Table 2). All analyses were performed using JMP®11 for Windows (SA, Institute Inc., Cary, NC, USA).

**Fig. 1:**
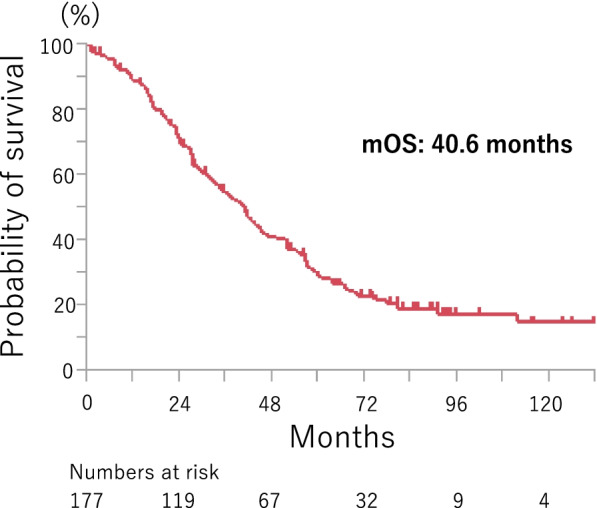
Kaplan–Meier curves of OS Survival data in the total study population

**Fig. 2:**
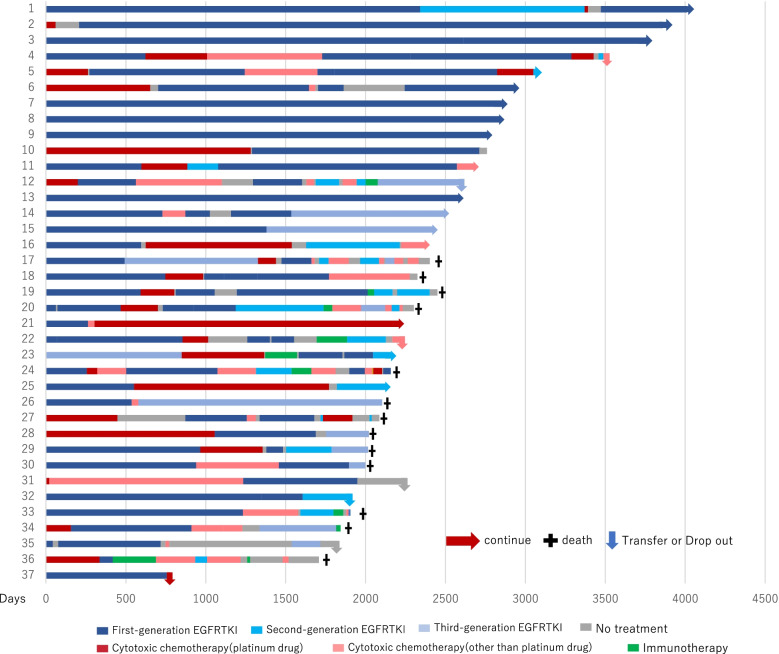
Swimmer plot of *EGFR*-mutated 5 years or more patients. 1–37 designates each patient. Blue indicates EGFR-TKI; red, cytotoxic chemotherapy; green, immunotherapy; and gray signals no treatment.

**Table 2:** Results of univariate and multivariate analysis of overall survival (Cox’s proportional hazard mode)

	**N**	**Univariate analysis**		**Multivariate analysis**	
		**HR(95% CI) **	***P* value**	**HR(95% CI) **	***P* value**
Sex					
Female	95	1	0.387	1	**0.0082**
Male	82	1.2(0.83–1.6)		1.8 (1.2–2.8)	
Age, year					
< 75	141	1	**0.047**	1	**0.0099**
≧75	36	1.5(1.00–2.3)		1.8 (1.2–2.9)	
Smoking history					
Current or former smoker	79	1	0.16	1	0.0721
Never smoker	93	1.3(0.90–1.8)		1.5 (0.96–2.2)	
ECOG PS					
0–1	135	1	**0.0002**	1	**0.0008**
2–4	21	2.6(1.6–4.4)		2.7 (1.5–4.7)	
Driver mutation					
* ALK*-Rearranged	22	1	0.0602	1	**0.0148**
* EGFR*-Mutated	155	1.7(0.98–3.1)		2.1 (1.2–3.8)	
Stage					
Post operative recurrence	70	1	**0.0007**	1	** < 0.0001**
Stage III or IV	107	1.9(1.3–2.7)		2.3 (1.5–3.4)	
Presence of brain metastasis					
No	132	1	**0.0009**	1	**0.0003**
	45	1.9(1.3–2.8)		2.1 (1.4–3.2)	

## Results

### Clinical characteristics of patients

All 177 included patients started their first chemotherapy between December 10, 2008, and September 30, 2015. There were 155 patients with *EGFR* mutations and 22 with *ALK* rearrangements. The median follow-up time was 36.9 months (range, 0.5–131.4) in the total cohort. The median follow-up time were 35.5 months (range, 0.5–131.4) with *EGFR* mutations and 51.8 months (range, 0.8–131.4) with *ALK* rearrangements. The median follow-up time was 89.2 months (64.6–131.4) in overall surviving patients. The median follow-up time were 89.4 months (range, 70.8–131.4) with *EGFR* mutations and 82.8 months (range, 64.6–131.4) with *ALK* rearrangements in survival patients. At the time of database lock, of the *EGFR*-mutated NSCLC patients, 17 patients (10.9%) were alive, 17 (10.9%) had been lost to follow up, and 121 (78.0%) had died. On the other hand, six patients (27.2%) were alive, three (13.6%) were lost to follow-up, and 13 (59.0%) had died among those patients with *ALK* rearrangements. Thirty-seven patients (23.8%) with *EGFR* mutations and nine (40.9%) with *ALK* rearrangements survived more than five years. In the patients with an *EGFR* mutation, the median total duration of EGFR-TKI treatments was 18.09 (range, 0–128.61) months; for cytotoxic chemotherapy, it was 8.08 (range, 0–64.37) months; and for immunotherapy, it was 1.34 (range, 0–9.53) months. In the *ALK*-rearranged patients, the median total duration of ALK-TKI treatment was 31.36 (range, 0–101.87) months; for cytotoxic chemotherapy, it was 8.17 (range, 0–44.18) months; and for immunotherapy, it was 0.69 (range, 0–0.69) months.

The clinical and pathological characteristics of patients are shown in Table 1. The median age at the initiation of first-line chemotherapy was 65 years (range, 34–89). Percentages were higher for females (53.6% vs 46.3%) and never-smokers (52.5% vs 44.5%). One hundred and fifty patients (84.7%) had an ECOG PS of 0–1, and twenty-one patients (11.7%) had a value of 2 or more. In terms of histology, 93.2% of patients had adenocarcinoma. Seventy of 177 patients relapsed after surgery. Extrathoracic metastases were found in 113 patients (63.8%) and brain metastases were found in 45 patients (25.4%).

The median number of chemotherapeutic regimens was three (range, 1–14), the median number of molecular targeted drugs was two (range, 0–7), and the median number of cytotoxic chemotherapy was one (range, 0–7).

### Survival

In the whole cohort, median survival time (MST) was 40.6 (95% CI, 32.9 to 45.2) months, the one-year survival rate was 89 (95% CI, 84.4 to 93.7) %, the three-year survival rate was 54 (95% CI, 46.7 to 61.7) %, and the five-year survival rate was 28 (95% CI, 22.1 to 35.7) % (Fig. 1). The OS curve seemed to plateau after 72 months, and survival rate at this point was 20.3 (95% CI, 13.6 to 27.0)% in *EGFR*-mutated patients and 46.0 (95% CI, 24.1 to 67.8) % in *ALK*-rearranged patients. Median OS was 36.9 (95% CI, 29.6 to 43.5) months in *EGFR*-mutated patients and 55.4 (95%CI, 32.9 to 111.3) months in *ALK*-rearranged patients (Supplemental Fig. 1-A). Statistically significant differences in OS were observed in five subgroups with log-rank test. Variables associated with significantly longer OS were: age less than 75 years (*p* = 0.027) (Supplemental Fig. 1-B), ECOG PS of 0 or 1 (*p* = 0.0002) (Supplemental Fig. 1-E), postoperative recurrence (*p* = 0.0015) (Supplemental Fig. 1-F), absence of extrathoracic metastases (*p* < 0.0001) (Supplemental Fig. 1-G), and absence of brain metastases at baseline (*p* = 0.0009) (Supplemental Fig. 1-H).

The number of patient with *EGFR*-mutated patients for the each subtype of *EGFR* gene was 74 patients (47.7%) with Exon 19 deletion, 63 patients (40.6%) with Exon 21 L858R, four patients (2.6%) with Exon 18 G719A/C/S, and three patients with Exon L861Q (Supplemental Table 1). There was no significant difference in survival between 19 del, L858R and others groups (Supplemental Fig. 2).

The univariate analysis was performed using the Cox’s proportional hazards model. The univariate analysis showed five variables to be associated with overall survival: age (< 75 141/177 vs. ≧75 36/177; *p* = 0.047); ECOG PS (0–1 135/177 vs. 2–4 21/177; *p* = 0.0002); Stage (post operative recurrence 70/177 vs. StageIII or IV 107/177; *p* = 0.0007); brain metastasis (presence 45/177 vs. absence 132/177; *p* = 0.0009); extrathoracic metastasis (presence 113/177 vs. absence 64/177; *p* = 0.0002) (Table 2). The multivariate analysis was performed using the Cox’s proportional hazards model. Six variables were consistently associated with significantly longer OS: female sex, age under 75 years, ECOG PS of 0 or 1, *ALK*-rearranged, postoperative recurrence, and absence of brain metastasis (Table 2).

### Subsequent clinical course in patients who survived five years or more

The clinical and pathological characteristics of patients surviving five years or more are shown in Table 3. Of 155 patients with *EGFR* mutation, 37 patients (23.8%) were alive for longer than five years. On the other hand, of 22 patients with *ALK* rearrangement, nine (40.9%) were alive for longer than five years.

**Table 3:** Clinical characteristics of patients who have survived over 5 years

Characteristics	All		*EGFR*-Mutated		*ALK*-Rearranged	
	*N* = 46	%	*N* = 37	%	*N* = 9	%
Age						
Median (range), years	62 (35–80)		64 (50–80)		51 (35–74)	
Sex						
Male	19	41.3	14	37.8	5	55.5
Female	27	58.7	23	62.1	4	44.4
Smoking status						
Never-smoker	20	43.5	16	43.2	4	44.4
Current or former smoker	23	50	18	48.6	5	55.5
Unknown	3	6.5	3	8.1	0	0
Pack-years, median(range)	32(2–114)		32(2–114)		29.5(13–48)	
ECOG performance status						
0	33	71.7	27	72.9	6	66.6
1	10	21.7	8	21.6	2	22.2
2	0	0	0	0	0	0
> 2	2	4.3	1	2.7	1	11.1
Unknown	1	2.1	1	2.7	0	0
Tumor histology						
Adenocarcinoma	42	91.3	34	91.8	8	88.8
NSCLC, NOS	1	2.1	0	0	1	11.1
Unknown	3	6.5	3	8.1	0	0
Stage						
Relapse	25	54.3	22	59.4	3	33.3
III	3	6.5	2	5.4	1	11.1
IV	18	39.1	13	35.1	5	55.5
Presence of extrathoracic metastases						
Yes	13	28.2	9	24.3	4	44.4
No	33	71.1	28	75.6	5	55.5
Presence of brain metastases						
Yes	5	10.8	2	5.4	3	33.3
No	41	89.1	35	94.5	6	66.6

**Fig. 3:**
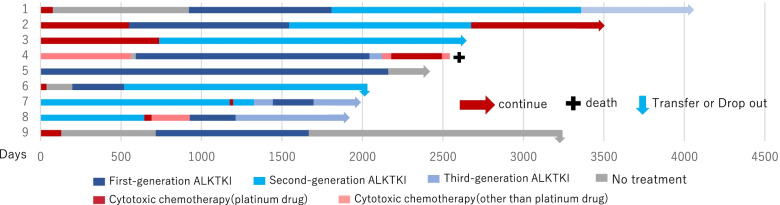
Swimmer plot of *ALK*-rearranged 5 years or more patients. 1–9 designates each patient. Blue indicates ALK-TKI; red, cytotoxic chemotherapy; and gray signals no treatment.

Figure 2 shows a swimmer plot of patients with *EGFR* mutations who have survived more than five years since their first chemotherapy; 1–37 designates each patient, as thirty-seven patients were alive for more than five years. In five patients, disease had been stable with just one EGFR-TKI, and in two, disease had been stable with two EGFR-TKIs. In the other patients, not only EGFR-TKIs, but also cytotoxic chemotherapy or immunotherapy were used to control their disease. In cases with long-term survival, EGFR-TKIs tended to be used for a longer period than the other agents, and some cases survived for 10 years or more with longer EGFR-TKI use. Figure 3 shows a swimmer’s plot of *ALK*-rearranged patients who have survived more than five years since their first chemotherapy; 1–9 designates each patient, as nine patients were alive for more than five years. Cases 1, 2, 4, and 9 were treated before the launch of ALK-TKI, and cytotoxic chemotherapy was used as first chemotherapy. As cases 7 and 8 were treated after the launch of alectinib, this was used as their first chemotherapy. In case 3, the disease was stable with a second generation ALK-TKI. In cases 5 and 9, disease was stable even after cessation of ALK-TKI.

## Discussion

As described in the introductory section, the discovery of driver oncogenes and the development of their specific inhibitors have improved the survival of patients with advanced NSCLC. ICIs were also found to be effective for the treatment of advanced NSCLC, and as they can suppress cancer progression for a longer time, a “tail-plateau” can be seen in Kaplan–Meier OS curves. There is no evidence that molecular targeted agents have the potential to cure lung cancer or to suppress cancer progression for a long time like ICIs. In the adjuvant setting, there is a phase 3 study, CTONG 1104, that compared gefitinib with vinorelbine plus cisplatin as adjuvant treatment for stage II-IIIA (N1-N2) *EGFR*-mutated NSCLC [20]. Adjuvant therapy with gefitinib in patients with early-stage NSCLC and *EGFR* mutation demonstrated improved disease-free survival (DFS) over standard-of-care chemotherapy, but the advantage of DFS did not translate into a significant difference in OS. IMPACT (WJOG6410L) is randomized phase 3 trial that compared gefitinib with vinorelbine plus cisplatin as an adjuvant treatment for stage II to III *EGFR*-mutated NSCLC after complete resection, but gefitinib cannot significantly prolong DFS and OS [21]. ADAURA is a phase 3 trial that compared osimertinib with placebo for three years in patients with completely resected, stage IB to IIIA, *EGFR*-mutated NSCLC. DFS was significantly longer among those who received osimertinib than among those who received placebo, but OS data are too immature to allow evaluation of any benefit here [22]. We could find no report on adjuvant ALK-TKI in *ALK*-rearranged NSCLC.

In this study, we investigated factors related to long-term survival with advanced *EGFR*-mutated and *ALK*-rearranged NSCLC. The MST of all patients was 40.6 months. In the subgroup analysis, the MST in *EGFR*-mutated NSCLC patients was 36.9 months and the MST in *ALK*-rearranged NSCLC patients was 55.4 months, tending to be longer in the latter. The five-year survival rate of *EGFR*-mutated NSCLC patients was 23.8%, and that of *ALK*-rearranged NSCLC patients was 40.9%. When multivariate analysis was performed on long-term survival factors, applying Cox’s proportional hazard model, six factors were significant. In descending order of hazard ratio these are: ECOG PS, postoperative recurrence, driver mutation, presence or absence of brain metastasis, sex, and age. And an approximately 2.7-fold difference was observed between the PS: 0–1 group and the PS: 2–4 group.

We also investigated the clinical course of long-term survivors after five years. There are some long-term survivors among patients with *EGFR* mutations and *ALK* rearrangements, and the survival curve appears to have a tail-plateau as shown in Supplemental Fig. 1-A. However, when the details of clinical courses were investigated as shown in swimmer’s plots, most of the patients had been treated with several types of chemotherapy. In five patients (cases 3, 7, 8, 9, and 13), their disease had been stable on just one EGFR-TKI, and in two patients (cases 1 and 15), their disease had been stable on two EGFR-TKIs (Fig. 2). In some cases, the use of ALK-TKIs was continued for more than five years. In case 3, the disease was stable with a second generation ALK-TKI (Fig. 3). In these cases, the disease may be cured or be in a “nearly cured state” with a tyrosine kinase inhibitor. However, because it is difficult to stop tyrosine kinase inhibitors in patients with advanced NSCLC, it is impossible to know whether the disease is cured. In two patients with *ALK*-rearranged NSCLC (cases 5 and 9), their disease was stable even after cessation of their ALK-TKI. This suggests an ALK-TKI for *ALK*-rearranged NSCLC has more potential to suppress the disease for a longer time like immunotherapy in patients at an advanced stage, and is effective for improving OS in an adjuvant setting than EGFR-TKI for *EGFR*-mutated NSCLC.

In Japan, osimertinib was approved and used as the first chemotherapy for *EGFR-* mutated inoperable or recurrent NSCLC in 2018. Since the subjects of this study were patients whose first chemotherapy was before osimertinib approval, from December 2008 to August 2015, 69% (108/155 patients) of patients received first- and second-generation EGFR-TKIs as their first chemotherapy. In this study, the OS of *EGFR*-mutated NSCLC was 36.9 months, which was longer than that in the IPASS study, at 21.6 months [23]. The reason may be that 16% (26/155) of patients took osimertinib. In addition, Jessica et al. reported that the OS in patients with *EGFR*-mutated lung cancer was 30.9 months. The five-year survival rate was 23.8%, which tended to be longer than previously reported [17].

In Japan, crizotinib was approved and used as the first chemotherapy for *ALK*-rearranged inoperable or recurrent NSCLC in 2012. Later, a phase 3 trial was conducted comparing pemetrexed or docetaxel as second-line chemotherapy and cisplatin or carboplatin plus pemetrexed as first-line chemotherapy with crizotinib, and a significant prolongation of PFS was observed [12, 13]. In addition, alectinib is an ALK inhibitor that selectively exhibits kinase inhibitory activity against ALK, and was approved in Japan in 2014. In this study, ALK-TKIs were used in cases subsequent to first chemotherapy, following the launch of ALK-TKIs.

When multivariate analysis was performed on long-term survival factors, applying Cox’s proportional hazard model, six factors (ECOG PS, postoperative recurrence, driver mutation, sex, age and presence or absence of brain metastases) were significant. Park et al. and Lee et al. examined clinical features associated with survival in patients with *EGFR-* mutated advanced NSCLC treated with an EGFR-TKI and identified tumor burden, quantified by the number of metastatic sites, as one such feature [17, 24, 25]. Brain metastasis is considered to be a prognostic factor in lung cancer. Incomplete penetration of the drug into the CNS through the blood–brain barrier worsens the response to first-generation TKI treatment of the brain and meninges [26].

This research, however, is subject to several limitations. The first is that owing to the retrospective design, undefined biases may have been present and influenced the clinical outcome. The next is that the data collection and analysis were performed at a single center, and the patient sample size is small. Finally, the patients chosen for this study were those in whom initial treatment was started more than five years ago, and the standard treatment at the time was different from current therapy.

## Conclusions

There are long-term survivors among patients with *EGFR* mutations and *ALK* rearrangements, and the survival curve appears to have a tail-plateau. However, most patients were treated with several types of chemotherapy, and were on treatment at the last follow up. In two NSCLC patients with *ALK* rearrangement, their disease was stable even after cessation of ALK-TKI, suggesting that ALK-TKI for *ALK*-rearranged NSCLC has more potential to suppress the disease for a longer time like immunotherapy in patients with advanced stage disease, and is effective at improving OS in the adjuvant setting than EGFR-TKI for *EGFR*-mutated NSCLC.

## Supplementary Information

**Additional file 1:Supplemental Table 1. **Distribution of *EGFR* mutation.

**Additional file 2.** 

## Data Availability

The datasets used and analyzed during the current study are available from the corresponding author on reasonable request.

## Abbreviations

NSCLC: Non-small cell lung cancer
*EGFR*: Epidermal growth factor receptor
*ALK*: Anaplastic lymphoma kinase
OS: Overall survival
ECOG PS: Eastern Cooperative Oncology Group Performance Status Scale
