# Supplementary material for: Survival past five years with advanced, EGFR-mutated or ALK-rearranged non-small cell lung cancer—is there a “tail plateau” in the survival curve of these patients?
Source: BMC Cancer. 2022 Mar 25;22:323. doi: 10.1186/s12885-022-09421-7 (PMC8953392; doi:10.1186/s12885-022-09421-7)
Supplement: Supplementary file 1 — Additional file 1:Supplemental Table 1. Distribution of EGFR mutation. [file 12885_2022_9421_MOESM1_ESM.docx]

**Supplemental Table 1. Distribution of *EGFR* mutation**

|  | N=155 | % |
| --- | --- | --- |
| Exon 19 deletion | 74 | 47.7 |
| Exon21 L858R | 63 | 40.6 |
| Exon18 G719A/C/S | 4 | 2.6 |
| Exon21 L861Q | 3 | 1.9 |
| Others | 4 | 2.6 |
| Unknown | 7 | 4.5 |
