# Supplementary material for: Survival past five years with advanced, EGFR-mutated or ALK-rearranged non-small cell lung cancer—is there a “tail plateau” in the survival curve of these patients?
Source: BMC Cancer. 2022 Mar 25;22:323. doi: 10.1186/s12885-022-09421-7 (PMC8953392; doi:10.1186/s12885-022-09421-7)
Supplement: Supplementary file 2 — Additional file 2. [file 12885_2022_9421_MOESM2_ESM.pdf]

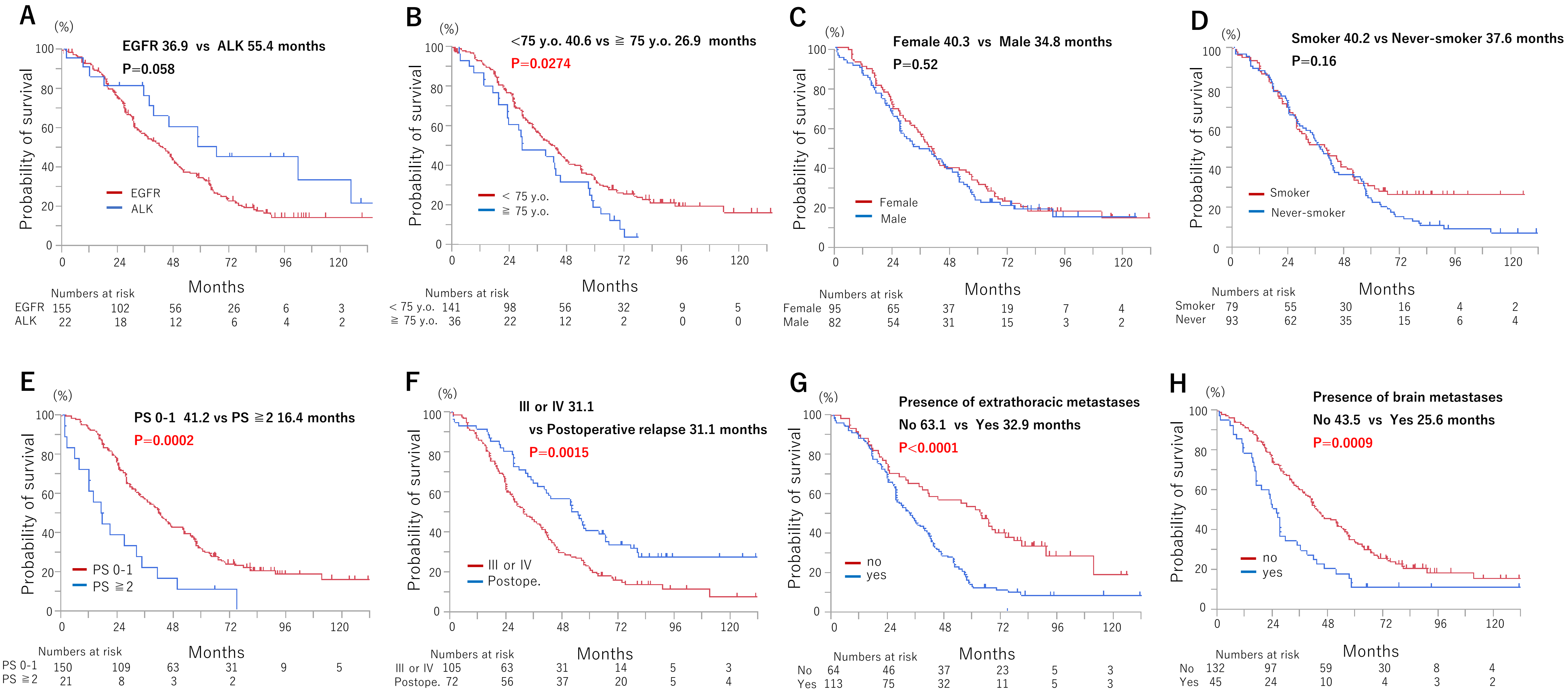

Supplemental Figure 1 Kaplan-Meier curves of OS in the subgroup of patients
(A) Generic mutation (*EGFR*-Mutated vs. *ALK*-Rearranged); (B) Age (≧75 vs. <75 years old); (C)Sex (Female vs. Male); (D) Smoking status (Current or former smoker vs. Never smoker); (E) ECOG Performance Status (<2 vs. ≧2); (F) Stage (Ⅲ and Ⅳ vs. Postoperative relapse); (G) Presence of extrathoracic metastases (Yes vs. No); (H) Presence of brain metastases (Yes vs. No)

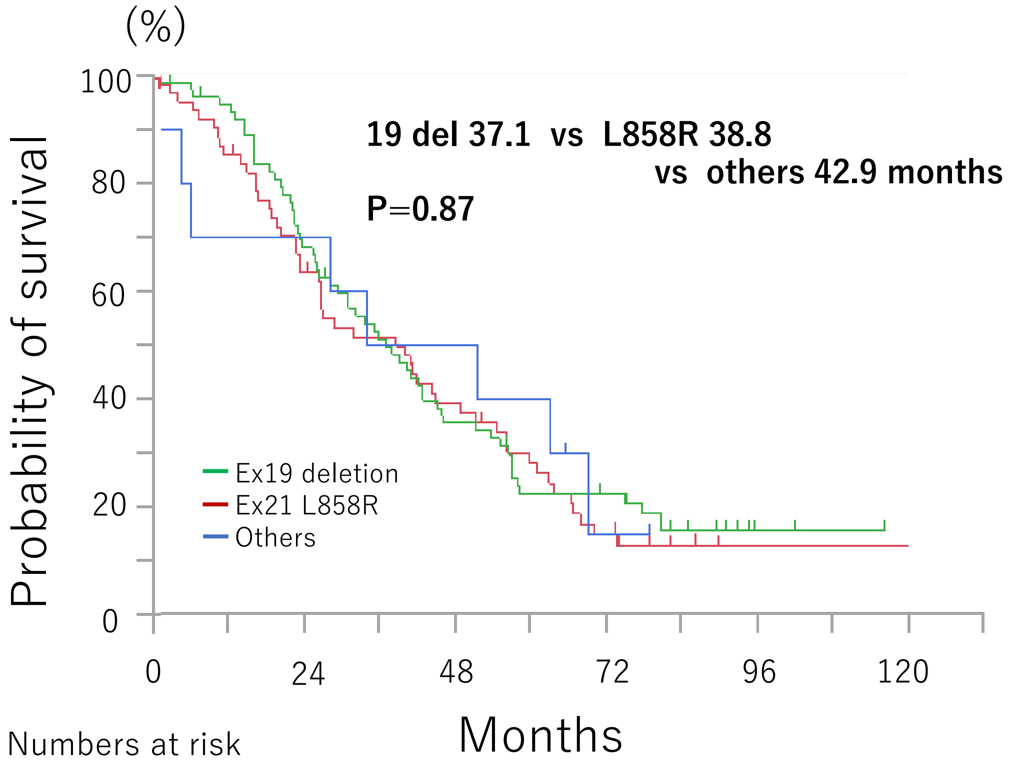

Numbers at risk						
19 del	74	51	26	15	3	1
L858R	63	39	22	9	2	2
Others	10	8	6	2	1	

Supplemental Figure 2 Kaplan-Meier curves of OS for the distribution of *EGFR* mutation
